# Condition Index, Reproduction and Feeding of Three Non-Obligatory Riverine Mekong Cyprinids in Different Environments

**DOI:** 10.21315/tlsr2020.31.2.8

**Published:** 2020-08-06

**Authors:** Natkritta Wongyai, Achara Jutagate, Chaiwut Grudpan, Tuantong Jutagate

**Affiliations:** Faculty of Agriculture, Ubon Ratchathnai University, Warin Chamrab, Ubon Ratchathani, 34190 Thailand

**Keywords:** Cyprinids, Lotic Environment, Lentic Environment, Life History

## Abstract

Condition index, reproduction and feeding of three non-obligatory riverine Mekong cyprinids namely *Hampala dispar*, *Hampala macrolepidota* and *Osteochilus vittatus* were examined. The samples were from the Nam Ngiep (NN) River and Bueng Khong Long (BKL) Swamp, which are the representative of the lotic- and lentic-environments, respectively. These two habitats lay in the same geographical area but on the opposite banks of the Mekong mainstream. The samplings were conducted between May 2017 and April 2018. There were 365 *H. dispar*, 259 *H. macrolepidota* and 298 *O. vittatus* samples in this study. The condition index of all three species were beyond 90% implying they can live well in both lotic and lentic environments. Reproductions of all three species were taken place in both environments with two peaks at the onset and end of rainy season. The samples from BKL showed early maturation than NN samples in all three (3) species. Feeding plasticity, though dominant by insects, was observed in *Hampala* spp., while *O. vittatus* can utilise any available detritus in both environments. Results clearly show that all the three selected non-obligatory riverine fish species can live very well in either lotic or lentic environments and imply that they can adjust themselves to reservoir environment.

**Highlights**

- The condition index revealed that three Mekong cyprinids *Hampala dispar*, *Hampala macrolepidota* and *Osteochilus vittatus* can live well in both lotic and lentic environments.
- Early maturations were found in all three species in the lentic environment.
- Various food items were found in stomachs of all three species, which indicate their high feeding plasticity.

## INTRODUCTION

Non-obligatory riverine fishes are fish species that can adjust their life history strategy to both lotic and lentic environments (Kruk & Penczak 2003; Kruk 2006). These fishes are reported to be more resistant to changes in flow regimes due to damming and dam operations (Kruk 2006). In contrast, obligatory riverine fishes, which are mostly migratory species, are at risk of extirpation after damming, due to the environmental shift, i.e. from lotic to lentic habitats, in association with the blockage of their migratory routes (Marmulla 2001; Han *et al.* 2008).

The potential impact of damming of rivers on disappearance of obligatory riverine fishes is of special importance for the Lower Mekong Basin (LMB), where 11 mainstream dams are planned and at least 71 projects in the tributaries are expected to be operational by 2030 (ICEM 2010). The losses of obligatory riverine fishes would cause reduction of fishery production and consequently a decrease in animal protein supply for the riparian people in LMB since fishes are a major protein source (Dugan *et al.* 2010; Ferguson *et al.* 2011; Ziv *et al.* 2012). The most comprehensive study by the Mekong River Commission revealed that there are 924 fish species in LMB, in which more than 200 species are migratory (MRC 2003; Valbo-Jørgensen *et al.* 2010). Meanwhile, the freshwater fish catch in this basin is about 2 million tonnes annually and accounted for almost 20% of the world’s freshwater capture fish (ICEM 2010).

A number of studies on life history traits of obligatory riverine fishes has been conducted because they are always being highlighted in the LMB, regarding consequences of damming of rivers (e.g. Baran 2006; Jutagate *et al.* 2007; Halls & Kshatriya 2009). On the other hand, the non-obligatory riverine species, which can behaviourally adapt to lentic environment, are less well studied, though they are capable of colonising impoundments after damming and become the main component of catches in the fisheries (Costa-Pierce & Soemarwoto 1990; Marmulla 2001). Many riverine species in family Cyprinidae can survive in either permanent or temporary lentic conditions (Valbo-Jørgensen *et al.* 2010; Malisa Ilyana *et al.* 2019). These behaviourally adapted cyprinids generally show short range longitudinal migration and migrate between the main river channels and connected floodplains, i.e. lateral migration (Baran 2006). Among these cyprinids, are *Henicorhynchus* spp., *Cirrhinus* spp., *Hampala* spp. and *Osteochilus* spp., which are reported to maintain populations in lentic conditions, as long as they are able to access flowing water periodically, i.e. during spawning season (Lim *et al.* 1999; Valbo-Jørgensen *et al.* 2010; Suvarnaraksha *et al.* 2011).

The presence of these behaviourally adapted species depends on how well they can live in the new habitat, and whether their reproduction is successful (Gray *et al.* 2000). The most common index to quantify the well-being of fish is the condition factor, which assumes that heavier fish of a given length are in better condition. This index can be established using the length-weight relationship (Froese 2006). In addition, difference in fish well-being is highly relevant to availability of food and reproductive condition (Froese 2006). This study aims to investigate the differences in well-being and life history traits, i.e. reproduction and feeding, of three non-obligatory riverine cyprinids *viz.*, *Hampala dispar, Hampala macrolepidota* and *Osteochilus vittatus*. These species were selected because they are the common fishes that found in many impoundments in the LMB and lack of comparative study on their lives in different environments. Sampling was conducted in two distinct habitats in Bueng Khong Long Swamp, Thailand and Nam Ngiep River, Lao PDR. The main testable hypothesis is that there is no cost for the three species to live either in a river or a swamp and, thus, they could well adapt to the man-made lentic environment such as reservoir.

## MATERIALS AND METHODS

### Study Sites

**Figure 1:**
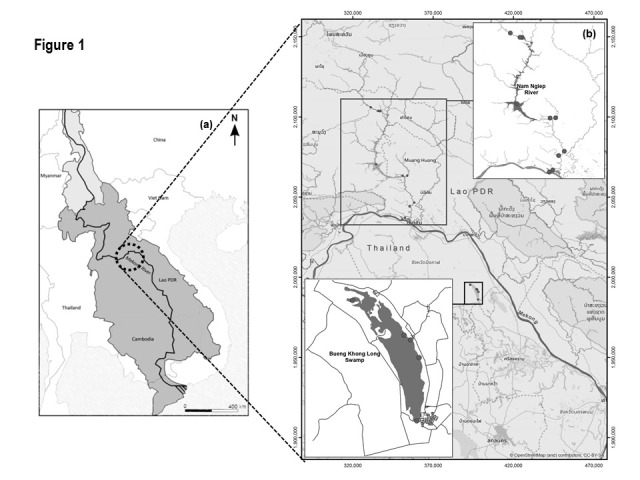
Location (a) and maps (b) of Bueng Khong Long (BKL) Swamp and Nam Ngiep (NN) River with the sampling stations.

The two sampling habitats (Fig. 1) for this study are the Nam Ngiep River in Bolikhanxay Province, Lao PDR, as representative of a lotic ecosystem, and Bueng Khong Long Swamp, Buengkan Province, Thailand, as representative of a lentic ecosystem. These two habitats are in the same geographical area but on the opposite banks of the Mekong mainstream.

The Nam Ngiep (NN) is a tributary of the Mekong of Lao PDR, originates from the Xiengkhuang plateau with an altitude of 1,050 m above mean sea level (N18.407705 and E103.605242 to N19.120453 and E103.321101). The river length is around 156 km with catchment area of 4,270 km^2^. The main tributaries are Nam Siam (120 km^2^) and Nam Chian (124 km^2^).

BKL Swamp (BKL; N18.044894 and E103.986012 to N17.959863 and E104.035206) covers the surface and catchment areas of 22.14 km^2^ and 59.8 km^2^, respectively. This swamp is the second largest Ramsar site of Thailand with an average water depth of 0.8 m. Inflow to the swamp *per se* is from many streams and channels, and excess water discharges into the Songkhram River and subsequently the Mekong River.

#### Sampling and data collection

The samples were collected monthly during May 2017 to April 2018, i.e. covered an annual hydrological cycle. Samples of each selected three species, i.e. *H. macrolepiota, H. dispar* and *O. vittatus*, were collected fortnightly from contracted fishermen (five) in each habitat Each fisherman used different mesh size of monofilament gillnets *viz*., 15 mm, 20 mm, 40 mm, 60 mm, 80 mm, 100 mm stretched mesh, for the sampling. The sizes of 6 different mesh widths were 25 m length with different height of 0.75 m, 0.6 m, 1.0 m, 1.5 m, 2.0 m and 2.5 m. respectively. Gillnets were set before sunset and retrieved in the next morning at each station. Each sample was measured in standard length (down to 0.1 cm) and weighed (down to 0.1 g). Subsequently fish samples were dissected in order to identify gender and to remove the gonads. Both ovaries and testes were then weighed (down to 0.1 g) and divided into five maturity stages (Nunez 2012), in which stage II and above were considered as matured. Only the ovaries were used in gonadosomatic index analyses.

Samples, with half-full to full stomachs, were dissected to study gut contents. Individual guts were preserved with 75% alcohol. Food items from each individual sample were identified under light stereomicroscope and divided into nine categories. Each food item category was then counted and weighed to the closest 1 mg.

### Data Analyses

The length-weight relationship of each species in each habitat was calculated by the length-weight regression (*LWR*) and the condition index (*CI*) was estimated by using the coefficients from *LWR* as in Equations 1 and 2, respectively (Froese 2006).

(1) $$W=aL^{b}$$

(2) CI=100×(WaLb)

where, *W* is the body weight of individual fish, *L* is standard length, *a* and *b* are coefficients. Difference in average *CI* between species was compared by habitat by mean of *t-*test.

The gonadosomatic index, *GSI* (Equation 3), was also calculated to track the changes in fish maturation by using the changes in weight of gonad, which only ovary was used in this study.

(3) $$GSI=\left(\frac{Gonad\_weight}{Whole\_body\_weight}\right)\times 100$$

The proportion of mature- to immature-fish (*P_i_*) at each length class (1 cm SL interval) of individual species was determined by the size at 50% maturity (*L_50_*: Equation 4)

(4) $$P_{i}=\frac{1}{1+e^{\left(S_{1}-S_{2}L_{i}\right)}}$$

where, *S_1_* and *S_2_* are the equation coefficients and *L_50_* was estimated as *S_1_/S_2_*. Meanwhile *L_25_* and *L_75_*, i.e. sizes at 25% and 75% maturity, were estimated as $\frac{\left(S_{1}-ln\;3\right)}{-S_{2}}$ and $\frac{\left(S_{1}+ln3\right)}{-S_{2}}$ respectively.

For each species in each habitat, importance of food item was evaluated by means of the index of relative importance (*%IRI*), using frequency of occurrence (*%O*), number (*%N*) and volume (*%V*) as in

(5) $$\%{IRI}_{i}=\frac{(\%N_{i}+\%V_{i})\times F_{i}}{\Sigma \left((\%N_{i}+\%V_{i})\times F_{i}\right)}$$

## RESULTS

### Length-weight Relationship and Condition Index

This study examined 922 individuals of three Cyprinids, i.e. 365 *H. dispar*, 259 *H. macrolepidota and* 298 *O. vittatus* both in BKL and NN (Table 1). The samples were available all year round in BKL. Standard length and weight of three species from two different habitats used for this study ranged between 7.5 cm–22.2 cm SL and 10.2 g–257.3 g respectively for BKL, and 7.3 cm–30.4 cm SL and 10.2 g–749.8 g respectively for NN. There is no statistical difference in average length and weight of the samples in each species between the two habitats (Table 1).

Values of exponent *b*, i.e. the coefficient *b* in Equation 1, of *O. vittatus* indicated a positive allometric growth, while *H. macrolepidota* showed a negative allometric growth, i.e. *b* < 3.0, for both habitats. A contrasting result of the allometry was found in *H. dispar*, which was positive in NN and *vice versa* in BKL. The average condition factor of fishes in BKL ranged 97%–101%, meanwhile it ranged 93%–100% in NN (Table 1). The *CI* of *H. dispar* and *O. vittatus* in BKL was significantly higher than NN (*P < 0.001*), meanwhile non-significant difference (*P =* 0.151) was found in *H. macrolepidota*.

### Reproductive Aspects

**Figure 2:**
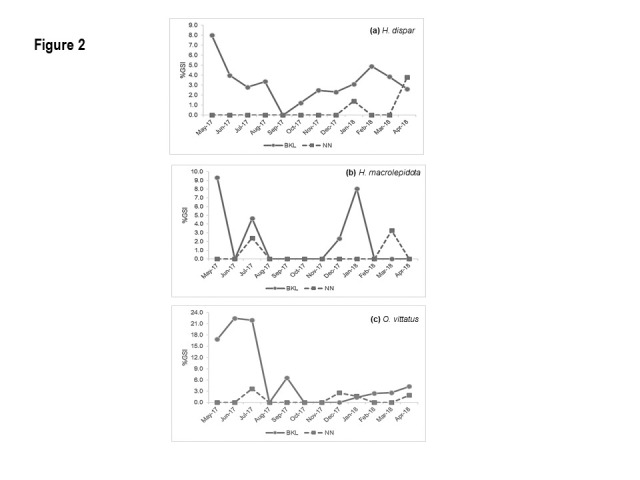
Changes in GSI of the three selected species during the studied period in both habitats.

A total of 157 samples was used for examining the GSI and 416 samples for examining the *L_50_*. The average GSI of *H. dispar* in BKL was markedly higher than NN (Fig. 2a). However, there were no samples of *H. dispar* in May and September in NN. It was obvious that *H. dsipar* in BKL matured almost throughout the year and the peaks GSI were observed in May (7.98 ± 2.35%). For NN, it showed the GSI only in January (1.41%) and April (3.80 ± 1.28%). Fluctuation trends of GSI of *H*. *macrolepidota* in both habitats seems to be similar but residents in BKL showed earlier maturity, i.e. a month prior to those in NN (Fig. 2b). Peaks in GSI of *H*. *macrolepidota* were observed for January (8.06%) and May (9.32%) in BKL and in July (2.41%) and March (3.26%) for NN. Maturity of *O. vittatus* in BKL occurred almost year-round and was clearly higher than in NN*.* The GSI of *O. vittatus* in BKL started increasing in January (1.39 ± 0.41%) and peaked in June (22.49 ± 4.71%). Two spawning periods were observed for *O. vittatus* in NN, according to the peaks of GSI (Fig. 2c), with the first around July (3.67 ± 2.36%), and the latter around December (2.58 ± 0.59%) to January (1.71 ± 1.09%).

**Figure 3:**
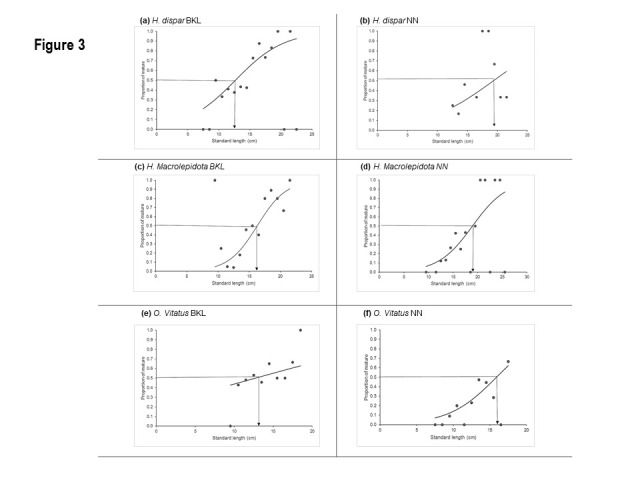
Scattered plots and fitted curves to the proportion of maturity of the three selected species during the studied period in both habitats.

Sizes of *L_50_* of the three species living in the lentic environment, i.e. BKL, were clearly smaller than those in the lotic environment, i.e. NN (Fig. 3; Table 2). The respective L*_50_* of *H. dispar*, *H. macrolepidota* and *O. vittatus* (cm SL) in BKL (12.66, 16.29 and 12.74 SL, meanwhile there were 19.33, 18.92 and 15.87 cm SL respectively for NN residents.

### Diets

**Figure 4:**
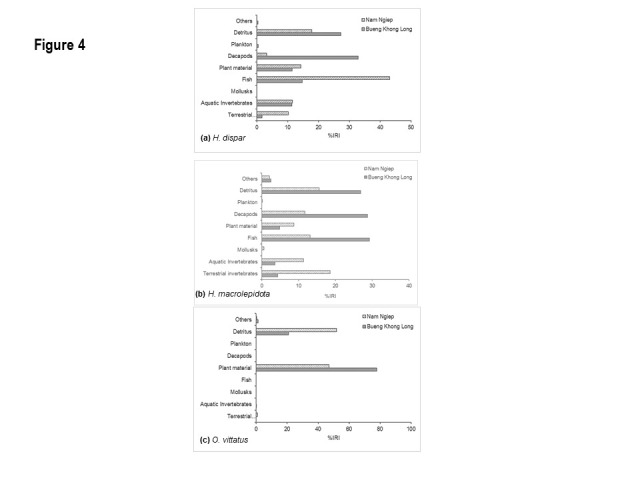
Index of relative importance (%IRI) of dietary items in the guts of the three selected species during the studied period in both habitats.

A total of 379 samples out of 473 with half to full stomach was used in the gut analyses (Fig. 4), i.e. 105 *H. dispar*, 130 *H. macrolepidota* and 144 *O. vittatus*. Decapods contributed the most in %IRI for *H. dispar* in BKL (33%), while it was fish (43% IRI) for those in NN. Interestingly the %IRI of decapods for *H. dispar* in NN was less than 5%. Contributions of aquatic invertebrates and plant materials in %IRI were quite similar for *H. dispar* in both habitats. Almost 10% IRI of *H. dispar* in NN was terrestrial invertebrates, but this was very low of *H. dispar* in BKL (Fig. 4a). For *H. macrolepidota* in BKL, fish, decapods (mainly shrimp) and detritus were almost equally important as the main dietary components, i.e. each contributed more than 20% IRI. A large variety of food items, *viz.* terrestrialand aquatic invertebrates, fish, decapods, plant materials and detritus, were all shown as important components of the diet for *H. macrolepidota* in NN, with more than 10% IRI each (Fig. 4b). Detritus and plant materials, e.g. grass, leaves and filament algae, made up the bulk of %IRI for *O. vittatus*. Both food items together represented more than 90% IRI for *O. vittatus*, with the diet in BKL dominated by plant materials and those in NN by detritus. Besides the two major food items, terrestrial insects were also found in NN samples, while there was also a small proportion of unidentifiable digested contents in BKL samples (Fig. 4c).

## DISCUSSION

The main purpose of this study is to provide evidence on types of adaptations of the three riverine species to either lotic or lentic environments, which are relevant to adaptations to man-made lentic habitats such as reservoirs.

The *b* values of length-weight relationship of all studied fishes were between 2.5 and 3.5 as expected in teleost fishes (Froese 2006). Froese (2006) also suggested that discussions on *b* values, between study areas, should refer to differences in condition between small and large individuals. The *b* values of *O. vittatus* in both habitats are positive, indicating that this fish becomes relatively deeper-bodied as it increases in length (Riedel *et al.* 2007). The negative allometric growth for *Hampala* spp. indicates it favours increase in length rather than in mass since their body shapes are more fusiform when they get larger (Makmur *et al.* 2014). Positive allometry found in *H. dispar* in NN showed the trend to isometric growth, i.e. *b* = 3, implying that, in this environment, this fish showed similar body form between the small- and large-individuals (Froese 2006). The condition index, i.e. the well-being, of fish varies between seasons, localities and years according to temperature, food availability, physico-chemical parameters and characteristic of habitats and genetic property as well as the condition of fish *per se* e.g. higher body weight of adult females during spawning season (Magnussen 2007; Zakeyuddin *et al.* 2012). Although a value of 100% or above in the condition index is optimal, indicating good health of individual fish in any particular habitat and situation. The overall average value found in this study of over 90%, for all studied species in both environments, also reflects favourable condition to live (Lloret *et al.* 2014)

The GSI can be used to determine the timing of the fish spawning season by evaluating gonad development (Manorama & Ramanujam 2017). It is recognised that most of tropical cyprinids are multiple spawners with prolonged spawning seasons, which are synchronised with changes in flow and seasonal rainfall patterns (Winfield & Nelson 1991). Meanwhile, the fluctuation in water level, resulting from meteorological and hydrological processes, is also the main trigger for spawning of fishes in the lentic environment (Hofmann *et al.* 2008). This can be clearly seen from the results with multiple peaks of GSI in all 3 studied cyprinids. Absence of *H. dispar* in NN samples during the May and September obfuscate any clear trend in GSI, though these two months, in particular May, are regarded as peaks in gonad development and main spawning periods for this fish in the rivers in Thailand and Cambodia as well as in the Mekong mainstream (Rainboth 1996; Termvidchakorn & Hortle 2013). It is then assumed that their absence from the catches is caused by moving to spawning habitats, i.e. dense vegetation floodplain, outside the fishing grounds, i.e. river channel, where the samples were collected. For *H. macrolepidota*, breeding can take place throughout the rainy season (Rainboth 1996). Although no clear continuous high GSI level was found in this study, the obvious peaks at the onset and end of rainy season were shown, though, not completely overlapping between the two habitats. For most cyprinids, the GSI starts to develop early in the year peaking during the rainy season, i.e. May to July (Rainboth 1996). This trend was clearly confirmed for *O. vittatus* in BKL as well as for NN, though it is difficult to infer due to the small peak during the rainy season. The two GSI peaks of *O. vittatus* in NN is similar to the *O. vittatus* stock in Phetburi River of Thailand, where the two peaks were in May to June and November to December, respectively (Setyaningrum *et al.* 2017).

Size at 50% maturity of three studied cyprinids from BKL is less than NN. Maturation schedules in fish are flexible and early maturity could be due to several reasons, including either the positive effects e.g. availability of feed and favourable feeding conditions (Yoneda & Wright 2005) or the negative effects, e.g. extreme environmental conditions (Blažek *et al.* 2013) and high fishing pressure (Hunter *et al.* 2015). More in-depth studies are needed to understand the main causes that make the studied cyprinids from BKL mature earlier than NN. However, based on the growth parameters obtained from FishBase (Froese & Pauly 2017) and the obtained size at 50% maturity, it can be said that these three cyprinids in both habitats could reach the maturity within 1+ years.

The gut content analysis is important in order to examine feeding pattern, feeding competition, structure of food web as well as the trophic level (Zacharia 2014). Most of the cyprinids are classified as omnivorous, feeding on invertebrates, detritus and vegetation (Wootton *et al.* 2000). Although most cyprinids are considered non-selective feeders, *Hampala* spp. are considered as diurnal predatory fishes in the ecosystem (Amarasinghe *et al*. 2008). In this study, major food items for both *Hampala* species also tended to be carnivorous fishes. Differences in dominant %IRI food items could be due to their availabilities. Interestingly, terrestrial insects dominated in stomach contents of *Hampala* spp*.* more in NN than BKL. Many studies in diet of stream fishes showed that numerous insects fell into the water and become prey for many riverine fishes and tend to become more dominant in particular during the flood season (Angermeier & Karr 1983; Baird 2007; Zakeyuddin *et al*. 2017). In contrast to *Hampala* spp, *O. vittatus’* diet was a mix between herbivorous and detritivorous, feeding exclusively on plant materials and decomposing plant and animal parts, similar to other studies on this fish (Yap 1988; Amarasinghe *et al.* 2008; Hanjavanit & Sangpradub 2012). It is, however, accepted that *Osteochilus* spp. can readily shift its diet and ingest crustaceans (Kakkaeo *et al.* 2004; Amarasinghe *et al.* 2008).

## CONCLUSION

Results of the study clearly show that all the three selected non-obligatory riverine fish species can live very well in either lotic or lentic environments, according to the wellbeing index. However, from a reproduction point of view, the GSI of the all three species in BKL were higher than NN indicating gonad development of fish is better in lentic conditions found in BKL with higher values in dry season. However, further studies are necessary to better understand the controlling factors. Meanwhile, dietary flexibility was clearly seen for *Hampala* spp., while *O. vittatus* can utilise any available detritus in the environment. Results also imply that these three species can adjust themselves to man-made lentic environments, i.e. reservoirs. Further studies are required into how well they can develop populations to support the fisheries in impoundments.

## Figures and Tables

**Table 1:** Length-weight relationship parameters for three cyprinids of BKL Swamp and NN River (*r*^2^: Regression Coefficient; C.I: Condition Index, A+: Positive allometric, A−: Negative allometric).

Habitat	Species	Samples	Mean length (cm) ± SD (range)	Mean weight (g) ± SD (range)	LWR	r^2^	Mean C.I. ± SD (range)	Growth type
BKL	*H. dispar*	315	14.5 ± 2.1 (7.5–22.2)	79.6 ± 36.3 (10.2–257.3)	W = 0.032L ^2.894^	0.933	101.0 ± 17.3 (68.6–363.7)	A−
	*H. macrolepidota*	132	14.2 ± 2.6 (9.8–21.0)	76.4 ± 45.7 (22.6–242.5)	W = 0.028L ^2.948^	0.969	99.0 ± 9.1 (75.6–119.8)	A−
	*O. vittatus*	174	13.7 ± 2.1 (9.5–18.3)	89.2 ± 49.3 (29.4–234.3)	W = 0.010L ^3.445^	0.957	97.4 ± 11.1 (58.9–150.8)	A+
NN	*H. dispar*	50	15.7 ± 2.7 (12.0–21.7)	95.7 ± 55.8 (36.5–276.4)	W = 0.019L ^3.073^	0.978	93.3 ± 7.4 (82.2–118.2)	A+
	*H. macrolepidota*	127	14.7 ± 3.0 (9.4–30.4)	87.5 ± 77.2 (14.1–749.8)	W = 0.037L ^2.846^	0.903	100.5 ± 13.5 (25.3–141.3)	A−
	*O.vittatus*	124	12.7 ± 2.0 (7.3–17.9)	65.4 ± 31.3 (10.2–182.0)	W = 0.020L ^3.155^	0.969	93.1 ± 9.3 (69.8–119.2)	A+

**Table 2:** Size at 25% (*L_25_*), 50% (*L_50_*) and 75% (*L_75_*) of the three selected species in BKL Swamp and NN River.

Species	Bueng Khong Long Swamp			Nam Ngiep River		

	*L_25_*	L_50_	L_75_	L_25_	L_50_	L_75_
*H. dispar*	8.38	12.66	16.95	13.05	19.33	25.60
*H. macrolepidota*	13.75	16.29	18.83	15.12	18.92	22.72
*O. vittatus*	NA	12.74	24.79	12.32	15.87	19.42

*Note*: The estimated *L_25_* of *O. vittatus* in BKL Swamp was too low (0.69), therefore it is marked NA-not applicable.
